# Optimizing the genetic prediction of the eye and hair color for North Eurasian populations

**DOI:** 10.1186/s12864-020-06923-1

**Published:** 2020-09-10

**Authors:** Elena Balanovska, Elena Lukianova, Janet Kagazezheva, Andrey Maurer, Natalia Leybova, Anastasiya Agdzhoyan, Igor Gorin, Valeria Petrushenko, Maxat Zhabagin, Vladimir Pylev, Elena Kostryukova, Oleg Balanovsky

**Affiliations:** 1 Research Centre for Medical Genetics, Moscow, Russia; 2 Biobank of North Eurasia, Moscow, Russia; 3 Vavilov Institute of General Genetics, Moscow, Russia; 4 Krasnodar State Medical University, Krasnodar, Russia; 5 Research Institute and Museum of Anthropology, Lomonosov Moscow State University, Moscow, Russia; 6 Institute of Ethnology and Anthropology of Russian Academy of Sciences, Moscow, Russia; 7 Moscow Institute of Physics and Technology, Moscow, Russia; 8 National Center for Biotechnology, Nursultan, Kazakhstan; 9 Federal Research and Clinical Centre of Physical-Chemical Medicine, Moscow, Russia

**Keywords:** Population genetics, Exome sequencing, Gene pools, Pigmentation, DNA markers, Eye color, Hair color, Appearance

## Abstract

**Background:**

Predicting the eye and hair color from genotype became an established and widely used tool in forensic genetics, as well as in studies of ancient human populations. However, the accuracy of this tool has been verified on the West and Central Europeans only, while populations from border regions between Europe and Asia (like Caucasus and Ural) also carry the light pigmentation phenotypes.

**Results:**

We phenotyped 286 samples collected across North Eurasia, genotyped them by the standard HIrisPlex-S markers and found that predictive power in Caucasus/Ural/West Siberian populations is reasonable but lower than that in West Europeans. As these populations have genetic ancestries different from that of West Europeans, we hypothesized they may carry a somewhat different allele spectrum. Thus, for all samples we performed the exome sequencing additionally enriched with the 53 genes and intergenic regions known to be associated with the eye/hair color. Our association analysis replicated the importance of the key previously known SNPs but also identified five new markers whose eye color prediction power for the studied populations is compatible with the two major previously well-known SNPs. Four out of these five SNPs lie within the HERС2 gene and the fifth in the intergenic region. These SNPs are found at high frequencies in most studied populations. The released dataset of exomes from Russian populations can be further used for population genetic and medical genetic studies.

**Conclusions:**

This study demonstrated that precision of the established systems for eye/hair color prediction from a genotype is slightly lower for the populations from the border regions between Europe and Asia that for the West Europeans. However, this precision can be improved if some newly revealed predictive SNPs are added into the panel. We discuss that the replication of these pigmentation-associated SNPs on the independent North Eurasian sample is needed in the future studies.

## Background

Predicting the eye and hair color from DNA became an important part of forensic genetic investigation. The genome-wide association studies [1–3] identified some key genes and sites within these genes which influence the pigmentation of the eye and hair color, as well as skin color [4, 5] phenotypes. These genes have been widely used for predicting pigmentation from genotype, mainly in the forensic context [6–8]. The most important sites have been included into HIrisPlex-S system [9–12]. Genotyping the 24 markers (SNPs and indel) [10] allows the rapid and reliable prediction of the eye and hair color (HIrisPlex system); additional 17 markers predict the skin color as well (HIrisPlex-S system).

The prediction has been shown to be reliable for populations of European descent and the system itself has been developed on European populations (mainly on Dutch). Its precision for populations from other regions of the world has not been tested extensively. Most non-European populations have brown eyes and dark hair only. However, some populations from border regions between Europe and Asia populations (for example, groups from Altai region in Siberia, some populations from the Caucasus) are also known to carry lighter eye/hair phenotypes and these populations do not exhibit close relation with West Europeans on the genome level [13]. Even populations from Ural region, being more related to West Europeans, than Caucasus and West Siberians, are nevertheless much more genetically distant from Dutch than populations of Ireland, Poland, and Greece, used for verification of the HirisPlexSystem [10, 11]. It is therefore possible that some Asian populations carry alleles of the pigmentation-related genes which are not included in HirisPlex-S but affects the appearance phenotypes in these populations. If this is a case, such additional alleles might be useful for eye/color prediction in these populations and have therefore practical importance for genetic forensic investigations in Russia, or when investigating individuals of Russian ancestry in Europe.

We aimed to estimate the precision of HirisPlex-S on different populations from North Eurasia, to search for new alleles within known pigmentation genes, and to estimate the impact of these alleles on the eye and hair color. To do so, we collected the DNA samples and photos from 300 individuals from indigenous communities from Russia and neighboring countries. The sampling covered European part of Russia, Caucasus region, Kazakhstan, and some populations from various parts of Siberia. We performed exome sequencing rather than genotyping to be able identify alleles which were not reported previously and therefore have not been included into the GWAS panels. As many key SNPs are known to be located in intronic regions, we developed the custom exome panel which includes both, exonic and intronic regions of the 53 genes and intergenic regions known to be involved in the pigmentation traits.

## Results and discussion

### Assembling the dataset

**Fig. 1:**
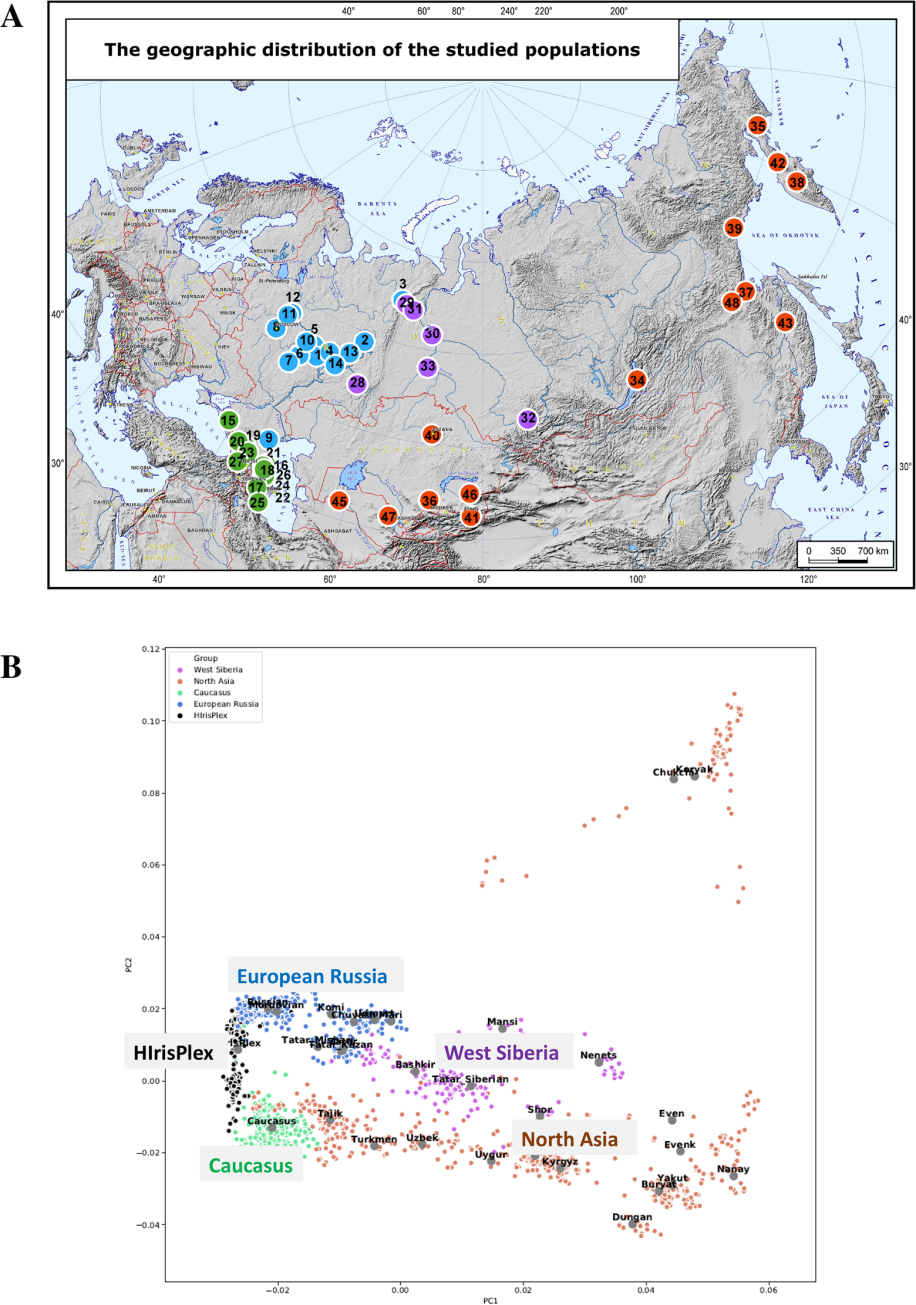
The studied populations. Panel **a**: The map of the studied populations. Numbers on the map refers to the following studied populations: 1 - Chuvashes, 2 - Komi Permyaks, 3 - Komi Zyrians, 4 - Mari Meadow, 5 - Mari Mountain, 6 - Mordvins Erzya, 7 - Mordvins Moksha, 8 - Russians, 9 - Russians Nekrasov’s Cossacs, 10 - Russians of Nizhny Novgorod region, 11 - Russians of Tver region, 12 - Russians of Yaroslavlsky region, 13 - Udmurts, 14 - Volga Tatars, 15 - Adyghe, 16 - Avars, 17 - Azeri, 18 - Dargins, 19 - Kabardinians, 20 - Karachays, 21 - Kumyks, 22 - Lezgins, 23 - Ossets, 24 - Rutuls, 25 - Talysh, 26 - Tsakhur, 27 - Turks Meskhetian, 28 - Bashkirs, 29 - Forest Nenets, 30 - Khanty, 31 - Mansi, 32 - Shors, 33 - Siberian Tatars, 34 - Buryats, 35 - Chukchis, 36 - Dungans, 37 - Evenks of Far East, 38 - Evens of Kamchatka, 39 - Evens of Okhotsk coast, 40 - Kazakhs, 41 - Kirghiz, 42 - Koryaks, 43 - Nanais, 44 - Tajiks, 45 - Turkmens, 46 - Uyghurs, 47 - Uzbeks, 48 - Yakuts of Far East. Panel **b**: The principal components plot for this study populations and for the populations used for HIris-plex-S developing/validation. HIris-plex populations are in black. Colors refers to the regional datasets present on the Panel A

We phenotyped 300 individuals from 48 populations of Russia and neighboring countries by identifying their eye and hair colors. Independent phenotyping by three experts and availability of photos for revisiting made the phenotyping reliable and reproducible. Populations were grouped into four regional datasets: European Russia, West Siberia, Caucasus, and North Asia; Fig. 1a presents the sampling locations and grouping into the regional datasets. In correspondence with the large area sampled, the regional metapopulations have contrasting genetic background. We performed the PC analysis of the populations included into this study to illustrate these findings (Fig. 1b). We note, that the populations on which the HIris-plex-S has been developed and validated (Dutch, Polish, Irish, and Greek) occupy the narrow zone on the “western” extreme of the PC plot, while populations present in our study, particularly North Asian, Caucasus and West Siberia are pronouncedly different from West Europeans and from one another. Thus, all downstream analyses were performed for each regional dataset and for the pooled dataset.

DNA samples from these 300 individuals were sequenced using the specially designed exome capture which included, in addition to the standard Roche exome capture, the intronic and intergenic regions known to carry pigmentation-related polymorphic sites (see Methods for details).

The combined dataset included phenotypic calls and genotypic calls for all individuals. Phenotypic calls included five categories of the hair darkness, three categories of the hair redness, and five categories of the eye darkness. Genotypic calls included genotypes of all polymorphic sites identified within the 53 genes and intergenic regions known to be involved in eye/hair pigmentation. The downstream analyses were performed on the subsets of this combined dataset.

### Validating the precision of HIrisPlex on north Eurasian populations

We started with estimating the precision of standard eye/hair prediction system in the newly phenotyped populations. From the combined dataset we extracted the phenotypic and genotypic calls for 24 SNPs included in the HIrisPlex-S. Then we predicted the eye and hair color from genotypes using the online HIrisPlex-S tool and compared the predicted phenotypes with the real phenotypes (Table 1). Table 2 presents the results for eye color prediction in different metapopulations (excluding the North Asia where the frequency of light eyes is low). We found (Table 1, Additional file 1) that the AUC value in the pooled North Eurasian dataset is only slightly lower than in the West/Central Europeans (particularly for the brown and red hair). However, when we analyzed the results for each region separately (Table 2), we found that performance of HIrisPlex-S panel for predicting eye color is lower for individuals from Caucasus region (AUC values are 0.83 and 0.78, for blue and dark eyes). Especially, the recall for blue eyes in Caucasus is significantly lower in comparison with the other North Eurasian regions - only 47% (Additional file 2). It might indicate that genes of the pigmentation metabolic pathways in the Caucasus populations carry allele spectrum somewhat different from that in Europe. When partitioning the dataset according to the phenotypic class (Table 1 and Table 2) we found that predicting the both, blue and brown eyes in Russian population is much less effective. In particular, the HirisPlex-S systems tends to misclassify blue eyes as brown.

**Table 1:** The AUC and accuracy of the eye color prediction using HirisPlex-S system and North Eurasian set of SNPs for the pooled North Eurasian dataset

	AUC				Accuracy		
	HIrisPlex-S on West/Central European populations	HIrisPlex-S on North Eurasian populations	North Eurasian SNPs (7 SNPs for eye and 11 SNPs for hair)	North Eurasian SNPs (36 SNPs for eye and 33 SNPs for hair)	HirisPlex-S on North Eurasian populations	North Eurasian SNPs (7 SNPs for eye and 11 SNPs for hair)	North Eurasian SNPs (36 SNPs for eye and 33 SNPs for hair)
Blue eye	0,94	0,93 (93)	0,96 (93)	0,9 (93)	0,86 (93)	0,83 (93)	0,97 (93)
Intermediate eye	0,74	N/A (6)	N/A (6)	N/A (6)	N/A (6)	N/A (6)	N/A (6)
Brown eye	0,95	0,93 (190)	0,86 (190)	0,97 (190)	0,86 (190)	0,79 (190)	0,98 (190)
Red hair	0,93	0,84 (18)	0,91 (18)	0,92 (18)	0,95 (18)	0,97 (18)	0,97 (18)
Blond hair	0,81	0,81 (40)	0,79 (40)	0,8 (40)	0,84 (40)	0,85 (40)	0,94 (40)
Brown hair	0,74	0,65 (70)	0,76 (70)	0,74 (70)	0,66 (70)	0,74 (70)	0,8 (70)
Dark hair	0,86	0,88 (156)	0,92 (156)	0,89 (156)	0,75 (156)	0,86 (156)	0,92 (156)

Note: number of samples in each phenotypic class is indicated in the parentheses

**Table 2:** The AUC and accuracy of the eye color prediction using HirisPlex-S set of SNPs for the regional North Eurasian datasets

	AUC			Accuracy		
	Caucasus region	West Siberia	European Russia	Caucasus region	West Siberia	European Russia
Blue eye	0,83 (15)	0,9 (17)	0,85 (60)	0,74 (15)	0,86 (17)	0,77 (60)
Intermediate eye	N/A (2)	N/A (1)	N/A (2)	N/A (2)	N/A (1)	N/A (2)
Brown eye	0,78 (38)	0,87 (26)	0,87 (32)	0,69 (38)	0,84 (26)	0,79 (32)
Red hair	N/A (0)	N/A (0)	0,81 (18)	N/A (0)	N/A (0)	0,88 (18)
Blond hair	0,77 (5)	0,58 (6)	0,75 (27)	0,84 (5)	0,79 (6)	0,72 (27)
Brown hair	0,41 (20)	0,55 (10)	0,6 (32)	0,41 (20)	0,65 (10)	0,61 (32)
Dark hair	0,77 (25)	0,81 (28)	0,76 (17)	0,53 (25)	0,77 (28)	0,79 (17)

Note: number of samples in each phenotypic class is indicated in the parentheses

### Eye and hair color prediction in north Eurasian populations: searching for new informative alleles. The general workflow

Our genetic data on the phenotyped individuals included the full sequencing of the pigmentation-associated genes and relevant intergenic regions rather than previously known SNPs only. Thus, we were potentially able to reveal the new informative alleles in the known genes. In total, we called 117,012 SNPs in the 53 genes and intergenic regions.

For eye color prediction we performed feature selection algorithms in order to obtain new informative alleles for North Eurasian populations for 4 datasets:

1. Pooled North Eurasian dataset
2. European Russia
3. Caucasus
4. West Siberia

For hair color prediction we used 5 datasets:

1. Pooled North Eurasian dataset
2. European Russia
3. Caucasus
4. West Siberia
5. North Asia

North Asian dataset was analyzed only for hair color prediction due to the fact for this region there is an observed variation in hair color while for eye color there is no such variation.

Each dataset has been divided in 60:40 ratio into training and test samples with preserving the percentage of samples for each class. For the pooled dataset we controlled that samples from different regions included in pooled dataset were split in the same proportion (60:40) to avoid region-related bias.

Feature selection procedure has been performed on the training dataset (Figure S2). Feature selection procedure consisted of applying three algorithms:

1. f_regression
2. mutual_info_regression
3. Lasso feature selection with different alphas (0.7, 0.5, 0.2, 0.1, 0.05, 0.01, 0.005, 0.001, 0.0005)

When analyzing the distribution of F score (from f_regression) and MI (from mutual_info_regression) the thresholds for the most effective features with highest scores were set for each dataset individually. When performing the Lasso feature selection we tested different choices of the alpha parameter. For each value of alpha we calculated r2 scores on training dataset for corresponding subset of SNPs that have non-zero coefficients.

Among these subsets we selected the most important ones according to obtained r2 scores for each dataset individually.

Based on results from three algorithms of feature selection all selected SNPs were combined in the top SNPs lists for each dataset.

In each top SNPs list, we selected SNPs which have the best predictive power. These SNPs formed best SNPs lists which we used to build a classifier. To select the best SNPs, we used the same scale as HIrisPlex-S classificator:

1. blue, intermediate and brown for eye color
2. red, blond, brown and dark for hair color

We considered these classes independent from each other and tried to build the classifier with the best power and the smallest SNPs set.

We used separate ranking systems for eye and hair color prediction to estimate the importance and prediction power of each SNP in order to narrow down the SNPs lists.

The performance of the best selected features was validated on the test dataset. To evaluate the quality of the model we calculated R2 score (coefficient of determination regression score function) (https://scikit-learn.org/stable/modules/generated/sklearn.metrics.r2_score.html), AUC score, precision, recall and accuracy metrics.

### Eye color prediction

#### Identifying the top SNPs in the pooled north Eurasian dataset

To identify the top SNPs associated with the eye color in our sample we applied three algorithms: f_regression (F score), mutual_info_regression (MI), and Lasso feature selection with different alphas (0.7, 0.5, 0.2, 0.1, 0.05, 0.01, 0.005, 0.001, 0.0005).

We analyzed F (f_regression) and MI (mutual_info_regression) scores distributions across the samples and selected the top 30 SNPs with the highest scores.

According to results from Lasso feature selection we decided to include in top SNPs list the most crucial ones - the ones having non zero coefficients for alpha = 0.5 (2 SNPs for ‘eye color’ dataset and 2 SNPs for ‘hair color’ dataset) and alpha = 0.2 (8 SNPs for ‘eye color’ dataset and 8 SNPs for ‘hair color’ dataset) - these SNPs carry the most prediction power according to r2 score values distribution over different alphas. We also included SNP sets for alphas 0.1, 0.01 and 0.005.

The final top SNPs list consisted of 256 SNPs (Additional file 3).

#### Narrowing the list of SNPs and building classifier for eye color based on it

We assigned to each SNP a score from 0 to 3. The score 3 is assigned only for SNPs from the pooled dataset top SNPs list because of the results made for that dataset are much more robust than for regional datasets (sample sizes for the regional datasets are present in the Additional file 4). The score 3 is assigned to SNPs that are in top 5 with highest F score or have coefficients more or equal to 0.1 in absolute value in Lasso models for alpha 0.2 or have non-zero coefficients in Lasso models for alpha 0.5. For the pooled sample the score 2 is assigned to SNPs that are in top 10 with highest F or MI scores or have non-zero coefficients in Lasso model for alpha 0.2. The score 1 is assigned to SNPs that have coefficients greater or equal 0.1 in Lasso model for alpha 0.005. To all other SNPs we assigned the score 0. All 36 SNPs with non-zero scores formed the best SNPs list and were used for the classifier.

The five SNPs had the highest score 3. Two of them were well-known eye color-causing SNPs (rs1129038 and rs12913832) while the remaining three have not been reported previously as powerful eye color predictive alleles.

#### Variation of the best SNPs list across geographic regions

The entire analysis performed for the pooled North Eurasian dataset has been repeated for the populations from the three following regions separately: European Russia, Caucasus, and West Siberia. For regional datasets the score 2 was assigned to SNPs that were in top 5 with highest F and MI scores or had coefficients more or equal to 0.1 in absolute value in Lasso model for alpha 0.5 or non-zero coefficients in Lasso model for alpha 0.7. The score 1 was assigned to SNPs that were in top 6 with highest F and MI scores or have coefficients non-zero coefficients in Lasso models for alpha 0.7 and 0.5. Additional file 5 presents the resulting best SNPs sets for all three regions. The comparison of the regional lists and the list for the pooled sample is present in the Additional file 6. In general, the set of best SNPs is stable across the regions: the SNPs with the highest scores are included in the most lists, while among the other SNPs there are both, identified within every region and region-specific. Further study on the additional phenotyped samples is necessary to replicate the significance of the region-specific SNPs.

The merged SNPs list was ranked by total score (as sum of all scores for 4 samples: Caucasus, West Siberia, European Russia, and pooled) (Additional file 6). Top 7 SNPs have the highest total score and occurred in more than one dataset, which is an additional confirmation that these SNPs have a strong predictive power (Table 3). Two of those SNPs (rs1129038 and rs12913832) are already included in HIrisPlex-S panel, while other five SNPs are new candidates for eye color predicting in the North Eurasian populations. We estimated the frequencies of these five SNPs in North Eurasian populations (Additional file 7). Each SNP was detected with polymorphic frequencies in every regional population, so these SNPs are common rather than rare ones.

**Table 3:** The list of 36 best North Eurasian SNPs for eye color prediction

SNP_ID	Caucasus Score	European Russia Score	West Siberia Score	Pooled Dataset Score	HIrisPlex-S	dbSNP RSID	Gene
**chr15:28356859_C_T**	2	2	2	3	rs1129038	rs1129038	HERC2
**chr15:28365618_A_G**	2	2	2	3	rs12913832	rs12913832;4745	HERC2
**chr15:28392261_G_A**	2	2		3		rs12898729	HERC2
**chr15:28410491_C_T**		2	2	3		rs12916300	HERC2
**chr15:28495956_A_G**		2	2	3		rs12912427	HERC2
**chr15:28562998_T_C**		1		2		rs1614575	HERC2
**chr20:39272620_A_G**		1		2		rs4812447	Intergene spacer
chr1:119406130_C_T				2		rs1779446	Intergene spacer
chr1:3331899_A_G				2		rs1999528	PRDM16
chr15:28145024_T_C				2		rs2871886	OCA2
chr15:28364059_A_G				2		rs7494942	HERC2
chr15:28380518_T_A				2		rs4778249	HERC2
chr15:28383565_T_C				2		rs7403279	HERC2
chr15:28513364_T_C				2		rs916977;4744	HERC2
chr15:28530182_C_T				2	rs1667394	rs1667394;4743	HERC2
chr15:28566122_A_G				2		rs751089833	HERC2
chr19:7570978_T_C				2		rs685034	C19orf45
chr3:189429301_G_T				2		rs6804480	TP63
chr6:45136347_G_A				2		rs1324530	SUPT3H
chrX:66405249_C_T				2		rs34191540	Intergene spacer
chr10:87576467_C_T				1		rs7923503	GRID1
chr14:92909309_T_C				1		rs12588868	SLC24A4
chr15:28419048_T_G				1		rs35946704	HERC2
chr17:9107969_G_A				1		rs17742781	NTN1
chr19:7578733_A_T				1		rs586243	ZNF358
chr3:189552236_T_C				1		rs7653443	MIR944
chr3:33035542_T_C				1		rs4586761	GLB1
chr3:33111182_T_G				1		rs72856153	GLB1
chr3:54251172_G_A				1		rs11283625	CACNA2D3
chr3:54636061_G_A				1		rs34983676	CACNA2D3
chr4:87847613_T_G		1		1		rs10022539	LOC100506746
chr4:87851083_C_T				1		rs72667724	LOC100506746
chr5:60947483_A_G				1		rs1501841	C5orf64
chr5:73959526_A_G				1		rs2454846	HEXB
chr6:45419110_C_G				1		rs2820339	RUNX2
chr7:42032565_C_T				1		rs2237427	GLI3

Indicated in bold - new SNPs which demonstrated the high prediction power for the eye color

Columns: SNP_ID – SNP ID in format: chromosome:position in GRCh37_allele 1_allele 2. Caucasus score, European Russia Score, West Siberia Score, Pooled Dataset Score – scores as described in section “Eye color prediction” for corresponding datasets

HIrisPlex-S – RS ID if used in HIrisPlex-S. Otherwise empty

dbSNP RSID – RS ID in dbSNP database

Gene – Nearest gene for this SNP

#### The north Eurasian SNPs set performance

**Fig. 2:**
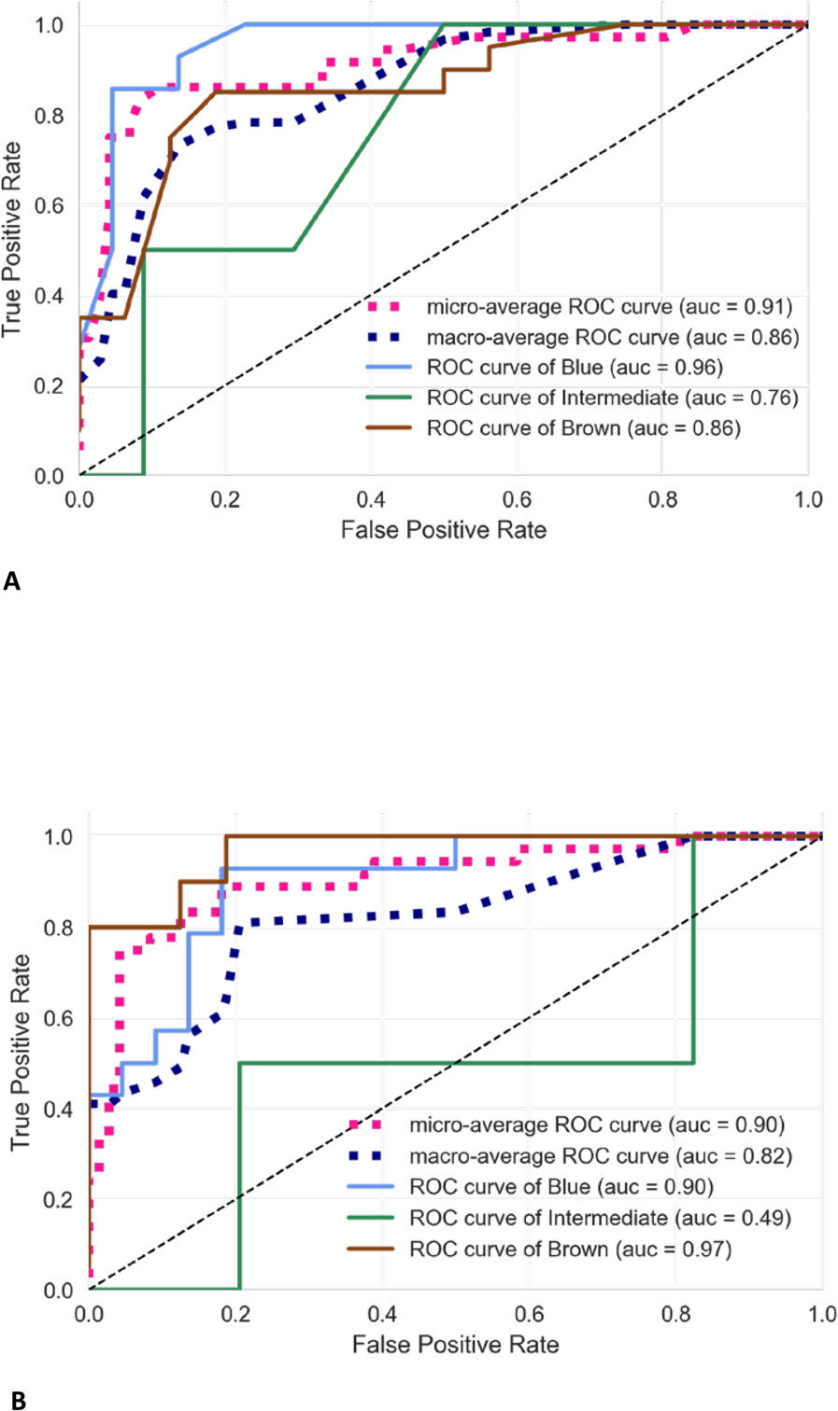
ROC-AUC curves for eye color prediction on North Eurasian dataset for three-grades scale. Panel **a**: results on the 7 SNPs set. Panel **b**: Results on the 36 SNPs

We estimated the performance of the SNPs which demonstrated the highest predictive power in our North Eurasian sample. The minimal set included 7 SNPs, two of which were previously included into the HIrisPlex-S panel. The optimal set included 36 SNPs which received the highest scores on the pooled North Eurasian dataset. We tested the classification performance of both sets of North Eurasian SNPs. Figure 2 presents the ROC curves and AUC scores for the prediction of three eye colors. The accuracy of 7 SNPs set is almost as effective as prediction based on the 41 HIrisPlex-S SNPs, while the set of 36 North Eurasian SNPs slightly outperforms 41 HIrisPlex-S SNPs on our sample (Fig. 2, Table 1).

### Hair color prediction

We performed the same feature selection analysis to find and evaluate top SNPs list for hair color prediction for pooled North Eurasian sample, which includes populations from the following regions: Caucasus, European Russia, West Siberia and North Asia.

We selected top 322 SNPs and narrowed the list to 33 best SNPs that have the strongest performance for 4-grade classification: red, blond, brown and dark hair color, the same scale as HIrisPlex-S (Additional file 8).

We assigned significance scores to select the minimum set of SNPs in following way:

1. The score 3 has been assigned to SNPs that are in top 5 with highest F or MI scores or have coefficients more than 0.05 in absolute value in Lasso models for alpha 0.2 or have non-zero coefficients in Lasso models for alpha 0.5
2. The score 2 has been assigned to SNPs in top 10 with highest F or MI scores
3. The rest SNPs of 33 best SNPs list have the score 1

We were able to detect the most powerful 11 SNPs that have the highest score (3), three of them are included in HIrisPlex-S panel (rs16891982, rs12913832, and rs1129038).

We checked the performance of the classifier based on 11 SNPs set and tried to estimate its ability to distinguish between 4 independent classes (the same as for HIrisPlex-S): red, blond, brown and dark hair (Additional file 9).

**Fig. 3:**
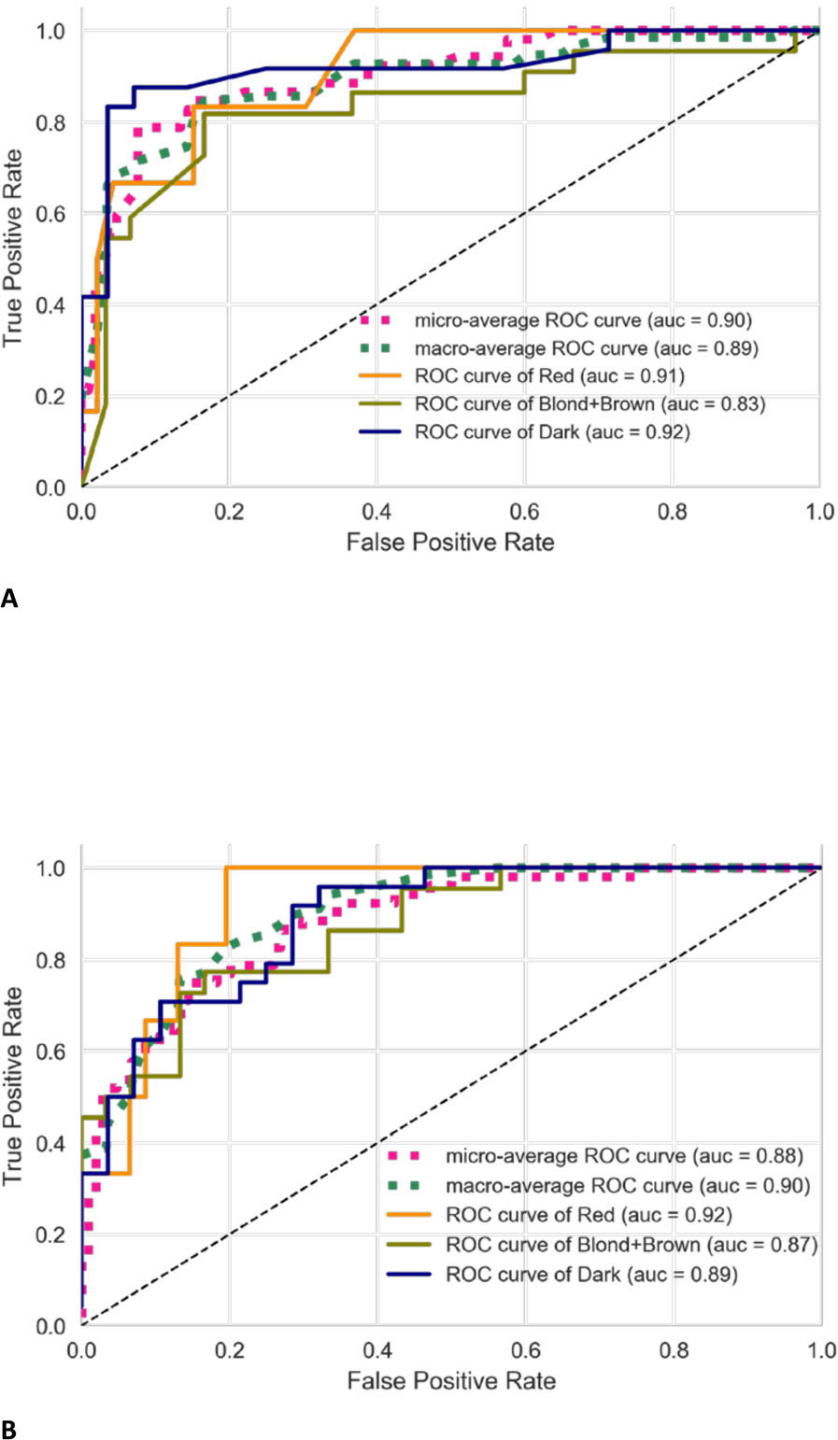
A. ROC-AUC curves for hair color prediction on North Eurasian dataset for the three-grades scale. Panel **a**: results on the 11 SNPs set. Panel **b**: results on the 33 SNPs set

Additionally, we tried to merge 2 classes of hair color - blond and brown - because algorithm does not have enough power to distinguish them, and checked the performance of selected SNPs for 3 grade scale. As we can see from the results (Fig. 3) the classifier performance improved significantly for both sets of SNPs: the most powerful 11 SNPs and 33 best SNPs.

### The new potentially informative SNPs

Our analysis identified five new SNPs which demonstrated the high prediction power for the eye color. These SNPs were revealed on the pooled North Eurasian sample and were replicated on the most regional subsamples. Four of these SNPs are located in *HERC2* gene, and one (rs4812447) is in intergenic region. *HERC2* (HECT And RLD Domain Containing E3 Ubiquitin Protein Ligase 2) gene belongs to the HERC gene family that encodes a group of unusually large proteins, which contain multiple structural domains. Genetic variations in this gene are associated with skin/hair/eye pigmentation variability [1, 14, 15].

### Limitations of the used approach

We analyzed the performance of the known pigmentation predictive SNPs and looked for the new SNPs in previously unstudied populations from different geographic areas. This regional-based approach allowed identify SNPs which are informative for the particular populations but made the sample sizes from each region quite limited. Therefore, we were not able to subdivide our sample into the training dataset and validation dataset – this would result in reducing sample sizes to numbers not allowing the statistically significant analysis. Therefore, our approach forced us to use the same dataset for SNPs discovery, building the classification model, and also for the validation, which might result in prediction overestimation. Therefore, the performance of our SNPs should be considered as an upper estimate, and the identified SNPs as candidate ones until verification on the independent sample in the future studies. Though stability of the top eye color predictive SNPs across geographic regions partly verifies the effectiveness of the newly identified predictive SNPs.

## Conclusion

We analyzed the gene-phenotype correlation in the populations from the border regions between Europe and Asia which carry light pigmentation phenotypes but have the contrasting genetic ancestries with the West Europeans. We replicated the effectiveness of the classical HIrisPlex-S panel for these previously unstudied populations, though the accuracy is slightly lower than for the West European groups the classifier has been developed on. Such decrease in accuracy might result from the population-specific SNPs which are present in North Eurasian populations but are rare in West Europeans and thus have not been included in the HIrisPlex-S panel. We analyzed the pigmentation genes and relevant intergenic regions in the phenotyped individuals and performed the association analysis between all identified polymorphic sites and pigmentation phenotypes. Note, that our target sequencing included not only the standard exome, but also intronic regions of the 53 pigmentation-related genes. Thus, the released dataset can be used for further population genetics or medical genetics studies, because it presents the exome variation in many indigenous groups which were previously studied by SNPs arrays only but not by the sequencing approach. As an additional by-product, the dataset allows to estimate the frequencies of the mutations with uncertain pathogenicity in the North Eurasian populations.

Our analysis of the pigmentation replicated the importance of the key previously known SNPs but also identified five new markers whose eye color prediction power on our North Eurasian dataset is compatible with the two major previously known SNPs. We note, that HIRisPlex-S recall for the blue eye phenotype is lowest in the Caucasus region, and our analysis identified a set of SNPs with prediction power specific for the Caucasus. We note, that all the SNPs revealed are candidate ones, as the same dataset (North Eurasian sample) has been used for both procedures: the feature selection and the classification. To avoid the overtraining effect, the replication of the new SNPs on the independent North Eurasian sample is needed in the future studies.

## Methods

### Populations studied

The dataset consisted of 300 samples from 48 local populations. Additional file 10 presents linguistic affiliation of these populations, while Fig. 1a indicates their geographic locations. The populations were geographically grouped into 4 major regions of North Eurasia (Fig. 1a, Additional file 10, Additional file 11).

To illustrate the genetic relations of these populations, we used the genome-wide datasets on the same ethnic groups available in the GG-base (www.gg-base.org) and run the principal components analysis using PLINK software (Fig. 1b). We also added into the analysis the populations used for developing and validating the HIris-plex-S system: Dutch, Irish, Polish, and Greek. As there were no data on Dutch populations in the GG-base, we used Northwest French and West Germans as a suitable proxy; this has had a minor impact on the plot, as PC has not identified much differentiation among the HIris-plex populations, as expected for West/Central Europeans.

### Phenotyping

The high-quality photos of members of indigenous North Eurasian communities were obtained during the field trips coordinated by the Biobank of North Eurasia [16]. The eye and hair color phenotypes were called based on these photos by three experts: two were physical anthropologists with deep experience in phenotyping, and the third was the specially trained geneticist. All the experts performed the phenotyping independently, and the cases when the calls were different became a subject of a thorough investigation until the consensus calls were achieved. We identified 99 individuals with light eyes and 187 with dark eyes, 128 individuals with light hair and 156 with dark hair, 76 individuals with red hair and 209 with not red hair. Additional file 11 presents the individual phenotyping calls including intermediate values.

### Library preparation and sequencing

Genomic DNA from both blood or saliva was extracted using an organic extraction method. List of genes and intergenic regions which can have potential polymorphisms associated with hair color and eye color traits was created based on detailed literature analysis [1, 14, 15, 17–25] including genome-wide association studies (GWAS) catalog (https://www.ebi.ac.uk/gwas/). For example, not exones only, but also introns of HERC2 and CACNA2D3 have been included into the sequencing capture. As a result, we developed the custom target sequencing panel which includes the 53 genes or intergenic regions. Fragmentation was performed by the Hydrodynamic Shearing System (Covaris). DNA fragments with ligated adapter molecules were selectively enriched by PCR, and then exons of genes were captured. The exome DNA enrichment was performed with custom SeqCap EZ Exome Plus Library Kit (Roche) with SeqCap Adapter Kit (Roche) and SeqCap HE-Oligo Kit (Roche) and sequencing libraries were generated using KAPA HyperPlus Library Preparation Kit (Roche), according to the manufacturer’s recommendations. Products were purified using the AMPure XP system (Beckman Coulter) and quantified using the Agilent high sensitivity DNA assay on the Agilent Bioanalyzer 2100 system. Sequencing was performed on an HiSeq 2500 sequencer (Illumina) with HiSeq SBS v4 250 Kit (Illumina) following the manufacturer’s recommendations and yielded 125-bp paired-end reads.

### Bioinformatics analysis

Raw data from high-throughput sequencing in fastq.gz format were aligned to hg19 reference human genome using bwa mem software. The resulting files in bam format were sorted and deduplicated using the SAMtools program package. Mutation calling was performed using freebayes software with filtration (quality (QUAL) > 40 & read depth (DP) > 5) of identified variants with vcffilter of vcflib program package. Annotation of variants was performed using SnpSift of snpEff program package. Databases dbSNP [26], dbNSFP [27, 28], ClinVar [29], 1000 Genomes Project [30], and ExAC [31] were used as information resources for identified variants. Samples with low genotyping rate have been excluded from further analyses (minimal genotyping rate is 90%), resulting in 286 samples dataset. Then polymorphisms in selected genes were analyzed and characterized for 286 samples.

### Prediction from Hiris-Plex-S SNPs

We called 41 polymorphic sites from HIris-Plex-S forensic panel in all 286 analyzed samples and calculated hair and eye color predictions for all samples using online tool of the Department of Genetic Identification of Erasmus MC (https://hirisplex.erasmusmc.nl). The predicted phenotypes were then compared with the true phenotypes, and the performance statistics were calculated for the pooled North Eurasian dataset and the regional datasets. Our five-grades scales have been converted into three-grades scales to make phenotypic call fully comparable with the HIrisPlex-S calls.

### Identifying the potentially informative SNPs for north Eurasian populations

Our dataset included 48 populations from North Eurasia. For eye color prediction we omitted the populations that are less polymorphic in eye color phenotypes (Additional file 11). This allowed us to achieve the better balance between different phenotypic classes. Populations which don’t have at least 4 grades of the our five-grades scale were eliminated from further analyses.

We ran feature selection algorithms in order to find most informative SNPs correlated with eye and hair color according to our 5-grade scales used for quantitative estimate of dark pigment in eyes (the highest value corresponds to the highest concentration of pigment) and hair (where ‘0’ is a red hair, ‘1’ is blond hair, and ‘4’ is the dark hair).

We considered our 5-grade scales (both for eye and hair color) continuous as they reflect the concentration of dark pigment and those classes are not independent.

Three feature selection methods were applied to select the most informative features associated with pigmentation traits for the pooled North Eurasian dataset and for each region separately.

Each dataset has been divided in 60:40 ratio into training and test samples using stratified K folds cross-validator that preserves the percentage of samples for each class.

Feature selection methods were applied to training dataset while the quality metrics for selected features were calculated using test dataset. The minimum set of SNPs with the most predictive power has been identified on test dataset. Also, we built classifier based on these final SNPs.

To evaluate the quality of the model we calculated r2 score (https://scikit-learn.org/stable/modules/generated/sklearn.metrics.r2_score.html), AUC, accuracy, precision and recall metrics using scikit-learn package [32].

### Feature selection algorithms

We used the three following algorithms for feature selection which are suitable for regression tasks (Additional file 12):

1. f_regression (https://scikit-learn.org/stable/modules/generated/sklearn.feature_selection.f_regression.html#sklearn.feature_selection.f_regression) - univariate regression test. Univariate feature selection works by selecting the best features based on univariate statistical tests. It uses linear model for testing the individual effect of each of many regressors. This is a scoring function to be used in a feature selection procedure.

This was done in 2 steps:

1. The correlation between each regressor and the target is computed, that is, ((X[:, i] - mean(X[:, i])) * (y - mean_y)) / (std(X[:, i]) * std.(y)).
2. It is converted to an F score then to a *p*-value

We selected only those features that has *p*-value< 0.01. Then we sorted them in F-score descending order. Features that have the highest F score values are considered the most promising and potentially informative features.

1. mutual_info_regression (https://scikit-learn.org/stable/modules/generated/sklearn.feature_selection.mutual_info_regression.html#sklearn.feature_selection.mutual_info_regression). It estimates mutual information (MI) for a continuous target variable. Mutual information between two random variables is a non-negative value, which measures the dependency between the variables. It is equal to zero if and only if two random variables are independent, and higher values mean higher dependency. The function relies on nonparametric methods based on entropy estimation from k-nearest neighbors’ distances as described in [33, 34]. Both methods are based on the idea originally proposed in [35].
2. L1- based feature selection -- Lasso technique (https://scikit-learn.org/stable/modules/generated/sklearn.linear_model.Lasso.html#sklearn.linear_model.Lasso). Linear models penalized with the L1 norm have sparse solutions: many of their estimated coefficients are zero. The Lasso is a linear model that estimates sparse coefficients. It is useful in some contexts due to its tendency to prefer solutions with fewer parameter values, effectively reducing the number of variables upon which the given solution is dependent.

It consists of a linear model trained with L1 prior as regularizer. The objective function to minimize is:

1 $$ \underset{w}{\min}\frac{1}{2{n}_{samples}}{\left|\left| Xw-y\right|\right|}_2^2+\alpha {\left|\left|w\right|\right|}_1 $$

The lasso estimate thus solves the minimization of the least-squares penalty with α||w||_1_ added, where α (alpha) is a constant, ||w||_1_ is the L1-norm of the parameter vector, X is training data and y is target values.

The parameter alpha controls the sparsity: the higher the alpha parameter, the fewer features selected. For our purposes we tested a range of alphas: 0.7, 0.5, 0.2, 0.1, 0.05, 0.01, 0.005, 0.001, 0.0005. The best features were found using the biggest alphas: 0.7, 0.5 and 0.2.

### Parameters for selecting the most significant features (SNPs)

#### The pooled dataset

To avoid the situation of finding SNPs associated with particular population of North Eurasia rather than with a phenotypic trait we excluded from analyses those geographic regions in which we didn’t find the variation in phenotype. Hence, for eye color prediction the final dataset included Caucasus, European Russia and West Siberia regions (Additional file 11), while the dataset for hair color prediction consisted of populations from all four regions - Caucasus, European Russia, North Asia, and West Siberia.

#### Identifying the top SNPs lists

Top of a few hundred SNPs most significantly associated with phenotypic traits has been chosen using the following thresholds (Additional file 12):

1. top 30 SNPs with highest F scores for f_regression
2. top 30 SNPs with highest MI scores for mutial_info_regression
3. SNPs with non-zero coefficients for Lasso models with alphas 0.5, 0.2, 0.1 0.01 and 0.005

#### Selecting the best SNPs from the top lists

The top lists included hundreds of SNPs, and to narrow down the lists we selected the best SNPs from each top-SNPs list (Additional file 12). We used corresponding thresholds to obtain these lists for both, eye and hair color prediction:

1. top 10 SNPs with highest F scores for f_regression
2. top 10 SNPs with highest MI scores for mutial_info_regression
3. SNPs with non-zero coefficients for Lasso models with alphas 0.5 and 0.2
4. SNPs with coefficients more or equal to 0.1 in absolute value for Lasso model with alpha 0.005

#### Regional datasets

To select best SNPs for each region we also performed 3 types of feature selection analyses and looking at the distribution of scores and considering the sample size for each region we set the following thresholds:

1. top 6 SNPs with highest F scores for f_regression
2. top 6 SNPs with highest MI scores for mutual_info_regression
3. SNPs with non-zero coefficients for Lasso feature selection with parameters alpha 0.7 and 0.5.

#### Building the classifier

For building the classifier we used a linear regression algorithm. We used genotypes for SNPs from best SNPs lists converted to values 0, 1 or 2 (2 for genotype ‘1/1’, 1 for genotypes ‘1/0’ or ‘0/1’ and 0 for ‘0/0’). Model was trained on the training dataset. For quality estimation we calculated r2 score, AUC, accuracy, precision and recall metrics on the test dataset.

## Supplementary information

**Additional file 1 Table S1.** Performance characteristics of North Eurasian SNPs sets and HIrisPlex-S panel on North Eurasian pooled dataset for eye and hair color prediction.

**Additional file 2 Table S2.** The performance of HIrisPlex-S panel on North Eurasian regional datasets.

**Additional file 3 Table S3.** Top SNPs list for North Eurasian pooled dataset for eye color prediction.

**Additional file 4 Table S4.** Sample sizes of different phenotypic classes in the regional datasets.

**Additional file 5 Table S5.** Best SNPs lists for regional datasets for eye color prediction.

**Additional file 6 Table S6.** Summary table of best SNPs lists for the North Eurasian pooled and regional datasets for eye color prediction.

**Additional file 7 Table S7.** Frequencies of the five new eye color predictive SNPs in North Eurasian populations.

**Additional file 8 Table S8.** Top SNPs list for North Eurasian pooled dataset for hair color prediction.

**Additional file 9 Figure S1.** ROC-AUC curves for hair color prediction on North Eurasian dataset for the four-grades scale. Panel A: results for the 11 SNPs set. Panel B: results for the 33 SNPs set.

**Additional file 10 Table S9.** The studied populations.

**Additional file 11 Table S10.** The analyzed North Eurasian samples.

**Additional file 12 Figure S2.** SNP selection scheme.

## Data Availability

The exome sequencing dataset used in this study has been deposited in European Nucleotide Archive database (https://www.ebi.ac.uk/ena) under PRJEB35224 (ERP118250) identification number of the study.

## Abbreviations

AUC: Area under ROC curve
ROC curve: Receiver operating characteristic
CACNA2D3: Calcium channel, voltage-dependent, alpha 2/delta subunit 3 gene
DNA: Deoxyribonucleic acid
GWAS: Genome-wide association studies
F: F-regression score
HERC2: HECT and RLD domain containing E3 ubiquitin protein ligase 2
MI: Mutual info regression score
PC: Principal component analysis
PCR: Polymerase chain reaction
R2 score: Coefficient of determination regression score function
SNP: Single nucleotide polymorphism
