# Supplementary figures and images for: Optimizing the genetic prediction of the eye and hair color for North Eurasian populations
Source: BMC Genomics. 2020 Sep 10;21(Suppl 7):527. doi: 10.1186/s12864-020-06923-1 (PMC7488246; doi:10.1186/s12864-020-06923-1)

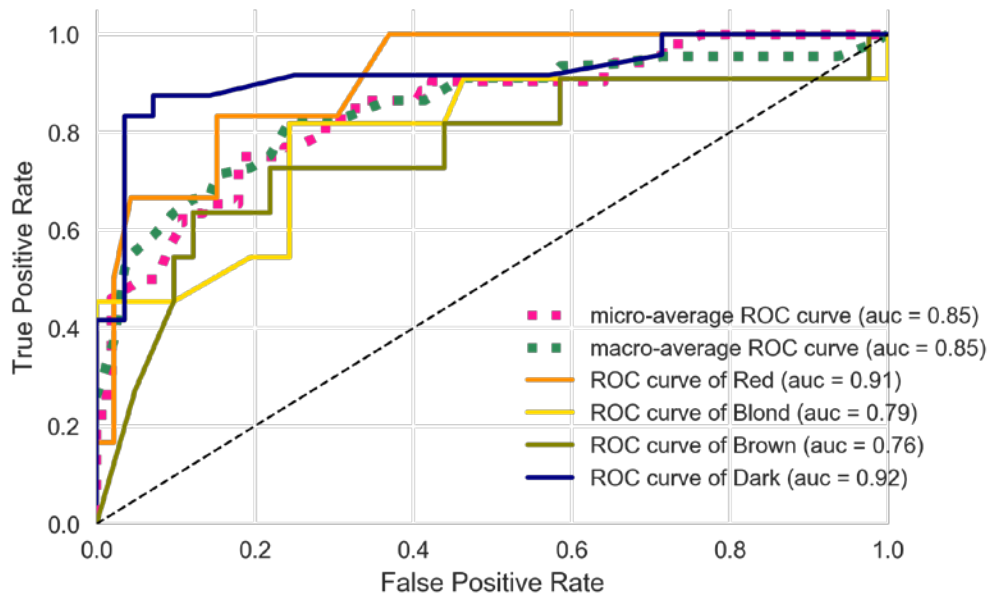

**A**

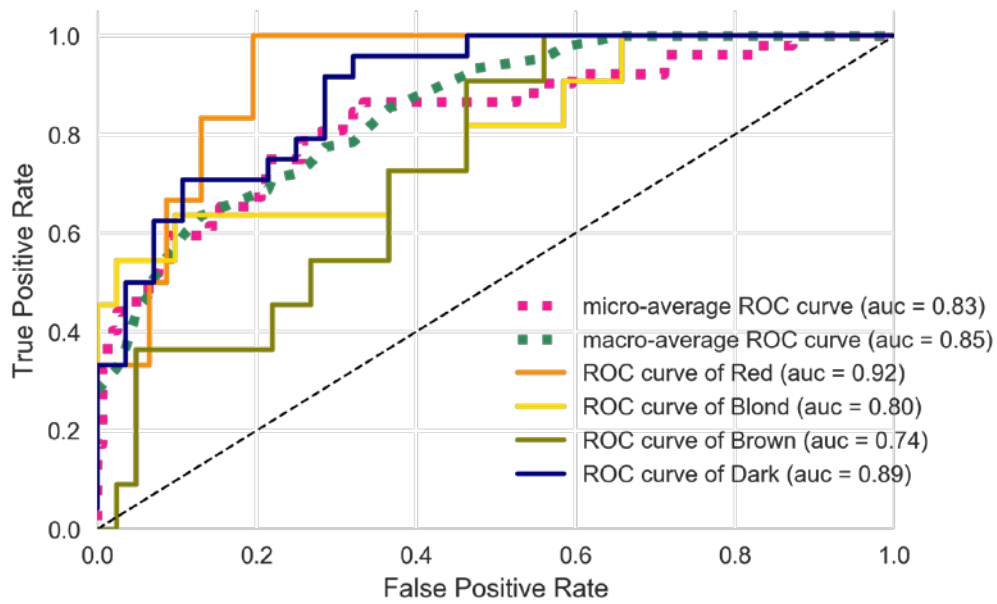

**B**

Supplement: Supplementary file 9 — Additional file 9 Figure S1. ROC-AUC curves for hair color prediction on North Eurasian dataset for the four-grades scale. Panel A: results for the 11 SNPs set. Panel B: results for the 33 SNPs set. [file 12864_2020_6923_MOESM9_ESM.pdf]

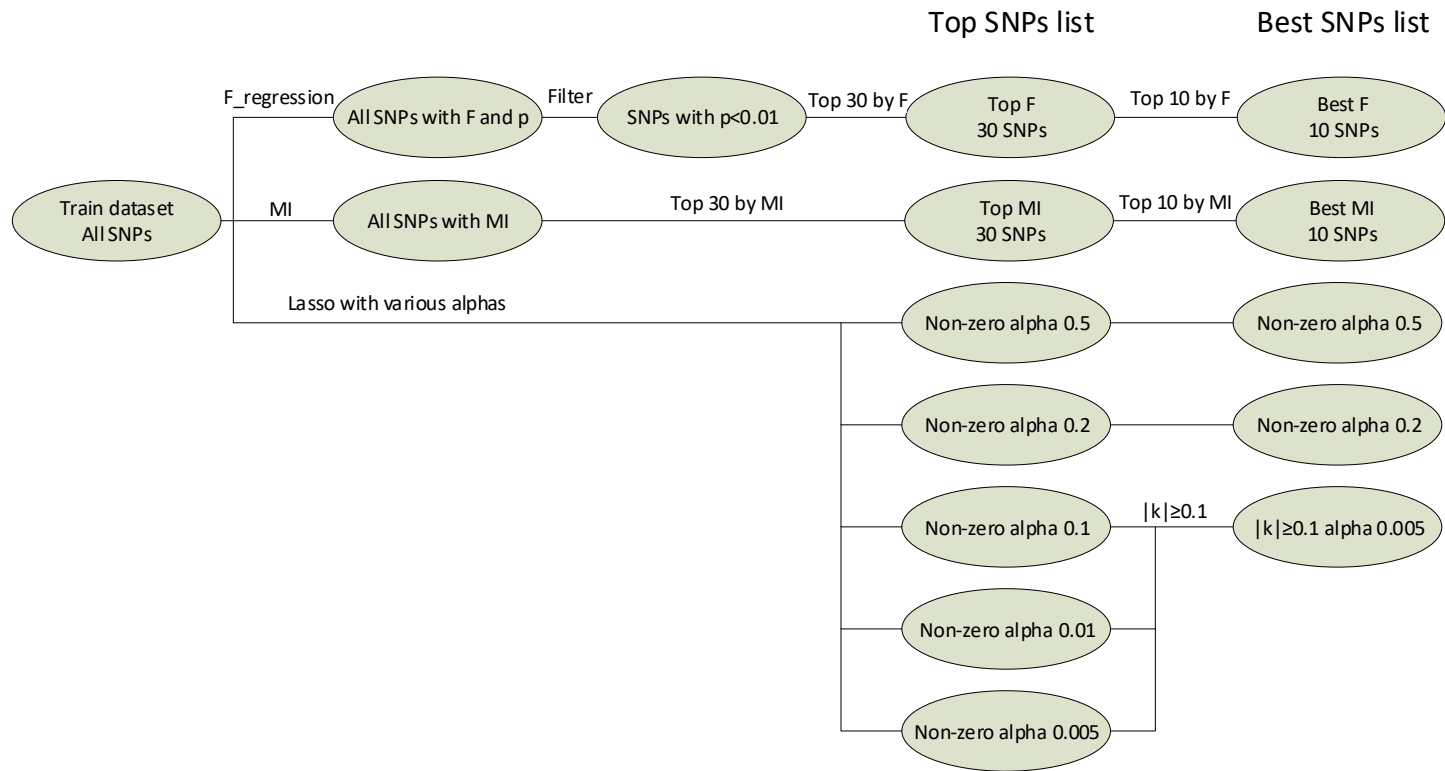

Supplement: Supplementary file 12 — Additional file 12 Figure S2. SNP selection scheme. [file 12864_2020_6923_MOESM12_ESM.pdf]
